# Homestead Law

**Published:** 1898-05-21

**Authors:** 


### Modern Sociology.

### HOMESTEAD LAW.

When a woman of the middle or upper classes marries, her father or other near relative does his best to secure her future welfare by a marriage settlement. By this the bridegroom settles on his wife a fixed income, if he has already in his possession property that will provide this, or insures his life in her favour, or settles on her the house they are to live in, if that is his property, or, at the least, his furniture is secured to her, not merely in the case of her husband's death, but in the event of his contracting debts which he cannot pay. This part of his property, settled on his wife, creditors cannot touch. No such precautions surround the wife of the artisan. She marries, content with her husband's promise to share all he has with her, and often enough with but little consideration of the probable responsibilities and troubles of married life. That women of that class or their friends will ever "bother with" the formalities of a marriage contract is most unlikely; indeed, many of them would think that it was paying but a poor compliment to the bridegroom to suggest that such a precaution will ever be necessary. Yet for no class is such a protection more desirable. Among the poor the illness of the husband, resulting in his death, may leave the wife literally without a penny when all the creditors are paid. Or a husband may, without his wife's knowledge, contract debts which he is unable to pay, and the poor furniture of the household may be sold to meet them. This is all the harder when, as is often the case, the furnishing of the home represents the wife's economy as much as the husband's labour.

To meet such contingencies among those who do not secure themselves and their children by marriage settlements, there exists in many of the American States what is called a "homestead law." The intention of this is to secure, up to a certain moderate amount, the household belongings against what is called the "diligence of creditors," and even against the landlord's claim for rent. Let the husband die a beggar or become a bankrupt, there still remains for his wife and family this provision—the absolutely necessary furnishing of the home. In some states the value of the amount thus protected is limited to £100, in others it is as much as £1,000. The latter sum seems excessive, and doubtless it is much in excess of the whole worldly wealth of many who profit by the law.

It might appear, on the face of it, that such a law as this forms a sort of wrong to creditors. They have supplied goods; why should they not be paid for them? But it must be remembered that, when such a law is in force, the creditor knows exactly where he stands. He knows that if the man who buys from him has not property beyond the £100, or whatever the sum may be that forms the limit of the "homestead law" of his state, he gives him credit at his own risk. This is really fairer than our own custom; for with us a shopkeeper may give credit to a considerable amount, misled by the apparent prosperity of a well-furnished house, and afraid to inquire, for fear of losing his customer, if this furniture is available for the payment of debts. As a matter of fact, everyone knows of cases in which creditors have lost heavily through giving credit on the strength of such a semblance of prosperity; finding out, only when too late, that house, furniture, insurance policy—in short, all available assets—had been settled on a wife. In America, under the homestead law, a man's credit has to be very good before he can obtain goods to any large amount. Here the revelations of the bankruptcy court often show how far beyond his means of paying a man who keeps up a big show of wealth can "plunge." Honest tradesmen would not lose by a law which said plainly, "Thus far, in seeking payment of your debts, you may go, and no further." It would, indeed, make cash payments the almost invariable rule.

But one cannot say that all creditors deserve commiseration. Who is it that gives long credit to the poor man? Is it the grocer, the baker, the butcher, anyone who purveys the absolute necessities of life? No. Occasionally, in a time when a strike or lock-out is on, or when for some other reason a familiar customer is out of work and penniless, one of these tradesmen may give goods, the payment of which is by no means secure. Sometimes they lose through this; but it is very rarely they who take action against the property of the debtor. It is rather the tallyman, the credit draper, who first persuades the Adam or Eve of the humble home—it is Eve whom he tempts most successfully—to buy his goods, and then uses all the force of the law to secure payment, when some unforeseen reverse of fortune makes such payment most difficult. The temptation which the draper's shop holds for every woman is intensified for the working man's wife. It takes, people say, some courage for any lady to pass such an establishment; but how much worse is it when your draper comes to your door, will not be refused an entrance, displays his wares whether you will or no, and, instead of talking of cash payments, assures his victim that some small sum paid weekly, something so trifling that she will never miss it, will purchase the article she fancies. True, before this article is paid for there will be something else, and the end will be that she will never be out of the tallyman's debt. And when misfortune comes, when the bread-winner dies or some other catastrophe happens, it is he who will be most insistent in demanding his pound of flesh. However much one may condemn a woman for taking goods on credit which she has little hope of ever being able to pay, we cannot sympathise greatly with the person who lures her into extravagance, and we incline to welcome anything that would tend to lessen his activity. And this a homestead law would do. Some years ago an inquiry was made on the subject, and one of these peripatetic drapers very naively said that such a law would ruin his business. He, and such as he, might be the poorer for it; but the mass of the people would be much the richer. A law which made it unprofitable for anyone to encourage the feminine weakness of extravagance might help greatly to this end. Men, too, as well as women, are apt to contract debts which they have small chance of being able to pay.

It is by no means necessary for the protection of the home that the limit up to which the homestead law protects should be as high as it is in America. Twenty pounds, or even ten, would in many cases cover the whole of the worldly possessions of a working man; but to have these secured to his widow and family in the event of his death would often give the latter the chance of keeping together, and being decently, if poorly, brought up, instead of lapsing, as many do on the death of the bread-winner, into pauperism and vagrancy. What a woman will do to keep her home together we all know; to what hopeless depths she may sink when homeless, and when it is impossible to keep her family by her side, we know also.

